# A kagome map of spin liquids from XXZ to Dzyaloshinskii–Moriya ferromagnet

**DOI:** 10.1038/ncomms10297

**Published:** 2016-01-22

**Authors:** Karim Essafi, Owen Benton, L.D.C. Jaubert

**Affiliations:** 1 Okinawa Inst Sci & Technol, Onna, Okinawa 904 0495, Japan

## Abstract

Despite its deceptive simplicity, few concepts have more fundamental implications than chirality, from the therapeutic activity of drugs to the fundamental forces of nature. In magnetic materials, chirality gives rise to unconventional phenomena such as the anomalous Hall effect and multiferroicity, taking an enhanced flavour in the so-called spin-liquid phases where magnetic disorder prevails. Kagome systems sit at the crossroad of these ideas. Motivated by the recent synthesis of rare-earth kagome materials and the progresses in optical-lattice experiments, we bring together an entire network of spin liquids with anisotropic and Dzyaloshinskii–Moriya interactions. This network revolves around the Ising antiferromagnet and ends on (ferromagnetic) chiral spin liquids with spontaneously broken time-reversal symmetry. As for the celebrated Heisenberg antiferromagnet, it now belongs to a triad of equivalently disordered phases. The present work provides a unifying theory of kagome spin liquids with time-reversal invariant nearest-neighbour Hamiltonians.

## Abstract (web summary)

Spin systems on the frustrated kagome lattice exhibit classical and quantum spin liquids. Here, the authors present an anisotropic nearest-neighbour model with time-reversal symmetry which incorporates all known spin liquids, as well as promising chiral phases, into a wider unified network.

## Graphical abstract

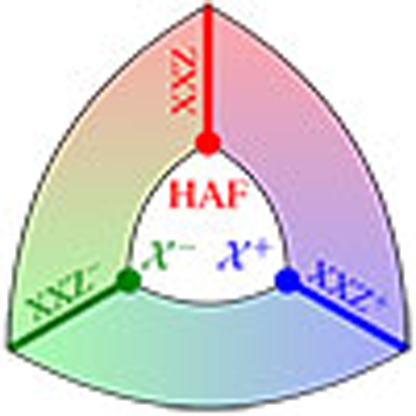

In condensed matter, competing interactions have proven able to stabilize extended phases where chirality could be encoded in the spin texture, that is, coming from the collective behaviour of spins. This spin-chirality is responsible for phenomena as varied as the anomalous Hall effect^1^,^2^,^3^, multiferroicity^4^ and possibly high transition temperature superconductivity^5^. In this context, kagome systems are fertile soil for exotic spin textures. Motivated by a growing number of materials^6^,^7^,^8^,^9^,^10^,^11^,^12^,^13^, the kagome lattice, whose name comes from a traditional Japanese woven bamboo pattern, has attracted the attention of chemists, experimentalists and theorists alike. The classical Heisenberg antiferromagnet (HAF) is a canonical example of order-by-disorder^14^, a counter-intuitive mechanism where order is induced by fluctuations. As for its quantum counterpart, it is one of the few models that has been confirmed to be a quantum spin liquid by a gamut of complementary approaches^15^,^16^,^17^,^18^,^19^. Recently, the kagome lattice has also been shown to support examples of the long-sought Kalmeyer–Laughlin chiral spin liquid^20^,^21^,^22^,^23^,^24^,^25^,^26^, a bosonic analogue of the fractional quantum Hall effect with anyonic excitations^27^.

Our present work sits at the frontier of these ideas of unconventional phenomena, spin liquids and chiral phases. We unveil a threefold mapping between kagome spin liquids, which is exact both at the classical and quantum level. This mapping brings into a general framework the well-known Heisenberg and XXZ antiferromagnets, together with a continuously connected network of models with Dzyaloshinskii–Moriya (DM) and anisotropic ferromagnetic (FM) couplings. All interactions are time-reversal 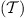 invariant and between nearest neighbours. For the end points of this connected network, 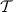 symmetry can be spontaneously broken in the classical ground state, giving rise to finite scalar chirality. The HAF maps onto a pair of systems characterized by FM pinch points in their structure factors, a signature of algebraic correlations constrained by an effective local flux conservation. Interestingly for quantum spin-1/2, the present work puts the Ising antiferromagnet at the centre of this connected network of quantum spin liquids, shedding a new light on the reluctance of this model to order^28^. On the experimental front, our phase diagram includes the Herbertsmithite compound ZnCu_3_(OH)_6_Cl_2_, which sits at the tip of an extended region of quantum disorder within linear spin wave theory. Our work is also motivated by the experimental possibility to explore a broad range of anisotropic interactions in the recently synthesized rare-earth kagome materials Dy_3_Ru_4_Al_12_ (ref. 12) and Yb_3_Ru_4_Al_12_ (ref. 13) and in optical lattices^29^,^30^.

## Results

### The model

**Figure 1:**
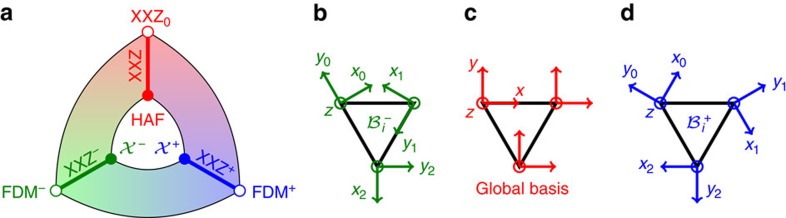
Threefold mapping of kagome spin liquids. We show the existence of an exact one-to-one mapping (**a**) made of local proper spin rotations (**b**–**d**) between the celebrated Heisenberg antiferromagnet (HAF) and two spin liquids 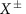 with opposite vector chirality given in equation (3). By tuning the anisotropy coupling *δ* of equations (5) and (6), our mapping directly extends onto the anisotropic XXZ model. Its chiral counterparts (named XXZ^±^) share the same extensive ground-state degeneracy as the XXZ model for *δ*<1, until the end point *δ*=−1/2 (FDM^±^), which belongs to the ferromagnetic model with Dzyaloshinskii–Moriya interactions, and where chirality becomes scalar. The local bases (**b**–**d**) are rotated by 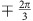 around the *z*-axis when moving from 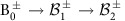. The *z*-axes are the same for all bases, which are right-oriented.

We focus on the nearest-neighbour Hamiltonian with anisotropic XXZ and DM interactions:


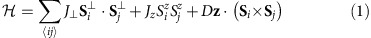


with in-plane components 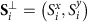 for a total number of *N* spins. We shall first consider classical Heisenberg spins of unit length 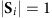, before considering the consequences of our theory onto quantum spins at the end of the Results section. The sublattice indices and Cartesian bases are given in Fig. 1. For perfect kagome symmetry, the DM vector is restricted along the unit vector **z**, orthogonal to the kagome plane^31^, using the clockwise convention for choosing the pairs of spins around the triangles.

To build a unifying picture of kagome spin liquids, one will take advantage of the rather precise understanding of the HAF (*J*_⊥_=*J*_*z*_=*J*>0 and *D*=0) that has been developed over the years. Among other points, its extensively degenerate classical ground-state manifold is locally constrained by a magnetization flux conservation. This constraint appears clearly if the Hamiltonian is re-written as


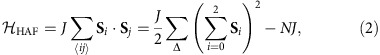


where the flux conservation takes the form of a null magnetization on all triangles Δ: 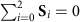.

### Exact threefold mapping

The peculiarity of the HAF lies in the form of its Hamiltonian given in equation (2). The idea of this paper is to find a one-to-one mapping (automorphism) of the spin degrees-of-freedom, which gives a Hamiltonian that can be re-written in the same form, while conserving the kagome symmetry and the spin unit-length, without imposing any spurious constraints.

To ensure the spin unit-length 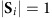, we consider local transformations **Γ** acting on each spin independently, that is, transformations from the global basis to a local one, 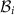: 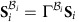 with 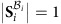. Then for the transformation to be non-trivial—that is, for 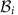 to be non-uniform—and to respect translation invariance, we attach one basis 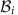 to each kagome sublattice. As a result, there are only two transformations respecting the space group symmetry of the kagome lattice. They are made of local proper rotations as illustrated in Fig. 1. They transform the HAF into the following models, which we name 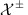



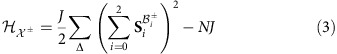



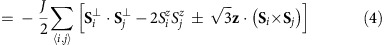


where 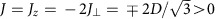. Since 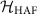 and 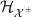 have the same form (see equations (2) and (3)), spin configurations connected by the one-to-one mappings 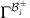 necessarily have the same energy in their respective Hamiltonians. Hence, the HAF, 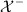 and 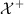 models have the same energy spectrum and thus the same extensive ground-state degeneracy. However, the spin rotation confers on them very peculiar signatures when probed magnetically.

**Figure 2:**
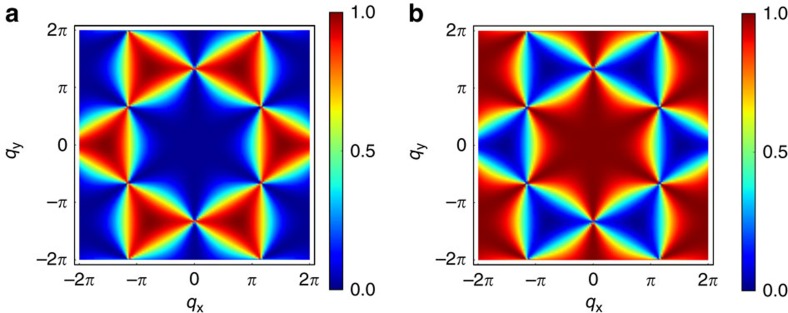
Structure factor of the Heisenberg antiferromagnet and 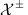 spin liquids. The Fourier transforms of the spin correlations have been computed using the method developed by Henley^71^ for Coulomb spin liquids in which the local constraints are enforced by a projection operator in reciprocal space. We have considered the planar components of the spin correlations 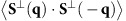, where **q**=(*q*_*x*_, *q*_*y*_) is the wavevector in Fourier space. ‘Pinch-point' singularities are formed in the centre of the Brillouin zones, characteristic of the local flux conservation discussed in equation (2). The structure factors clearly illustrate the underlying equivalence of the models, and the difference of their in-plane fluctuations; antiferromagnetic in the HAF (**a**) and ferromagnetic in the 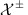 models (**b**). Only one figure is shown for the 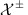 phases because they cannot be distinguished by the structure factor of the planar spin components. The colour scales are fixed from zero to maximum intensity of the structure factor on each figure.

The ground-state of the HAF, that is, the manifold of configurations with minimum energy, supports algebraic spin correlations^32^,^33^. In neutron scattering measurements, these correlations take the form of anisotropic diffuse scattering known as ‘pinch points' (see ref. 34 for a pedagogical review by Henley). As depicted in Fig. 2, pinch-point singularities are clearly visible in the structure factors of the 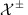 ground-state manifolds. The striking similarity of the HAF and 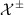 structure factors is actually a quantitative illustration of their underlying equivalence. However, because the planar spin components are, respectively, antiferromagnetically and ferromagnetically coupled in 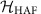 and 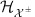, their collective fluctuations induce reversed spin correlations. This provides a noticeable example of pinch points induced by continuous FM fluctuations.

As *T*→0^+^, the 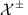 models are expected to undergo the same thermal order-by-disorder selection as the HAF^14^, with the additional flavour that the nematic/octupolar order^33^ now bears a finite vector chirality.

### A connected family of spin liquids

Spin-chirality takes multiple forms. The non-collinearity of spins is directly measured by the vector chirality **χ**_*ij*_=**S**_*i*_ × **S**_*j*_. For triangular units, one can further define a scalar chirality *χ*_*ijk*_=**S**_*i*_ · (**S**_*j*_ × **S**_*k*_), which is a measure of the solid angle formed by the three spins. Vector chirality comes from the spin current involved in the strong magneto-electric coupling of some multiferroics^4^ and the emergence of skyrmion excitations. As for scalar chirality, it can induce anomalous Hall effect when coupled to itinerant electrons^1^,^2^,^3^.

While vector chirality is intrinsically induced by the DM term, we do not expect any long-range scalar-chiral order in the 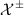 models since the HAF spin liquid does not break 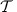 symmetry at finite temperature. It is thus tantalizing to see if, by taking advantage of the present threefold mapping, it were possible to tune the Hamiltonians and induce scalar chirality spontaneously.

**Figure 3:**
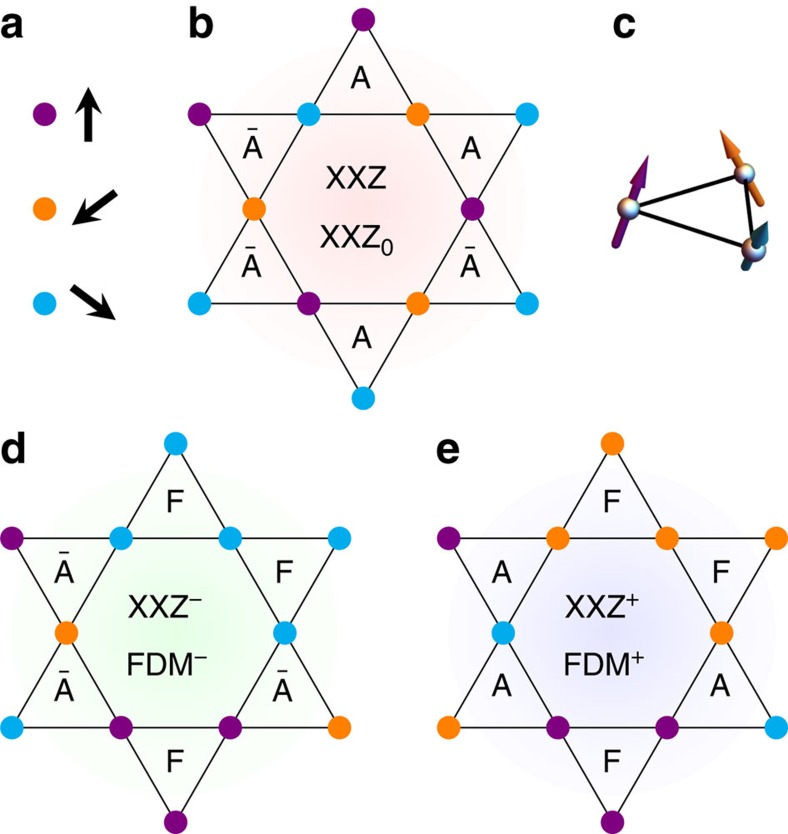
Three-colouring ground states. The classical ground-state ensemble of the XXZ model and its chiral counterparts XXZ^±^ are equivalent to the three-colouring problem, up to a global O(2) symmetry. The equivalence is transparent for the XXZ ground state (**b**) [35], where each triangle possesses the three possible spin orientations rotated by 2*π*/3 from each other, and *S*^*z*^=0. The colour code of the spin orientations is given in **a**. The two antiferromagnetic permutations *A*={
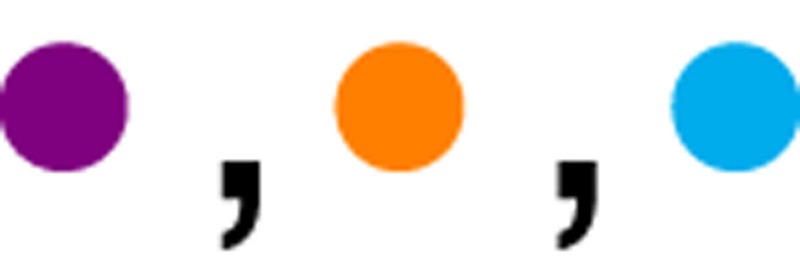
} and 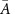
={
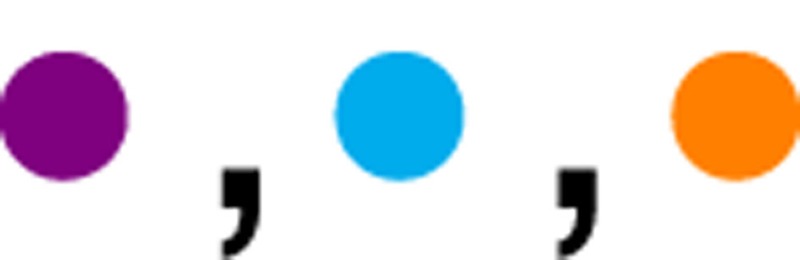
} are possible, giving a zero vector chirality on average. In this context, the apparition of vector-chirality in the XXZ^±^ ground states (**d**,**e**) is understood as the suppression of either the A or 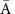 configurations in favour of a collinear state (F). The same scenario holds for the XXZ_0_ and FDM^±^ ground states where the spontaneous out-of-plane magnetization makes the chirality scalar (see equation (8)). An example of spin configuration with finite scalar chirality is given in **c**: the planar projection of the spins corresponds to configuration A.

Since our threefold mapping does not affect the *z*-axis (see Fig. 1), decreasing *J*_*z*_ has the same influence on the HAF and 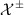 Hamiltonians: it tunes the HAF into the XXZ model


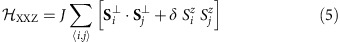


which is mapped onto what we name the XXZ^±^ models


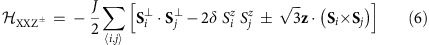


with *J*>0. This mapping is valid for all *δ* but for −1/2<*δ*<1, the XXZ ground state remains a sub-ensemble of the HAF one (*δ*=1) where all spins lie in plane (*χ*_*ijk*_=0). This ground state is equivalent to the three-colouring problem up to a global *O*(2) symmetry^35^, whose degeneracy is countable and extensive^36^. As illustrated in Fig. 3, the system is entirely paved with only two kinds of triangular configurations, A and 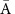, with opposite vector chirality^35^. The noticeable consequence of our mapping is that in the XXZ^−^ and XXZ^+^ ground states, the A or 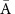 configurations are respectively replaced by a collinear state F with zero chirality; the resulting imbalance ensures finite and opposite vector chirality between the two XXZ^±^ ground states, while preserving their extensive degeneracy. From this point of view, it is interesting to think of the XXZ ground state as coming from the cancelation of positive and negative DM terms, once ferromagnetism has been taken out.

### Chiral spin liquids

On the other hand, for *δ*=−1/2 DM interactions become perfectly balanced by isotropic FM coupling


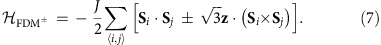


We name them the FDM^±^ models. As a consequence, for each triangle, both the DM induced^31^ and FM ground-state configurations minimize the classical energy


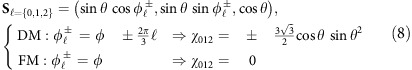


where the ± index distinguishes the two FDM^±^ models. With respect to the XXZ^±^ models where *θ* was imposed to be *π*/2, the global degeneracy of the FDM^±^ ground states is enhanced to *O*(3). Thus, while the **S**_⊥_ degrees-of-freedom conserves the character of a classical spin liquid, with the extensive degeneracy and algebraic correlations of the three-colouring problem, *S*^*z*^=cos *θ* can now take a finite uniform value, conferring a finite scalar chirality to any ground-state configuration with *θ*≠{0, *π*/2, *π*} (see equation (8)).

The threefold mapping transforms the FDM^±^ models back into a specific point of the XXZ model of equation (5) with *δ*=−1/2, which we shall refer to as XXZ_0_


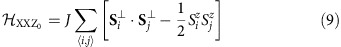


In the XXZ_0_ ground state, the scalar chirality persists on each triangle but vanishes on average. The enhanced global *O*(3) degeneracy remains. It is noteworthy that the end point value *δ*=−1/2 takes an elegant meaning along the XXZ^±^ lines, namely that the FM coupling becomes isotropic, which is hidden if only considering the XXZ model.

The emergence of scalar chirality in what is essentially a ‘simple' ferromagnet with DM interactions is quite remarkable, with a rich potential for unconventional phenomena. For example, the interplay between a chiral spin liquid and itinerant electrons is an up-and-coming topic^21^,^37^,^38^. Indeed, the FDM^±^ ground state is neither fully ordered like a solid or paramagnetic like a gas. In a pictorial way it is a magnetic liquid where strong correlations and fluctuations co-exist, which can then couple via double-exchange to another ‘fluid' made of itinerant electrons. While hopping on the scalar-chiral spin texture, the itinerant electrons pick up a Berry phase that might not only induce anomalous Hall conductivity^1^,^21^,^39^, but at the same time feedback into the strongly correlated spin texture to induce exotic magnetic order^21^,^40^,^41^,^42^. This feedback actually does not require scalar chirality and would also be pertinent to the XXZ^±^ lines.

It should be noted that given the large value of 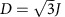, an experimental realization of the FDM^±^ models per se would arguably be difficult in solid state physics, but on the other hand, particularly promising for optical lattices. Indeed, the kagome geometry^29^ and spin anisotropy^30^ have been experimentally realized with ultracold atoms. There is also good hope that the active research on synthetic gauge fields might be able to produce synthetic DM interactions^43^,^44^,^45^, with the caveat that the DM vector should be out-of-plane here.

Last but not least, FM insulators with DM coupling have been studied in the context of magnon Hall effect, that is, where a transverse heat current is induced by a temperature gradient. It is intriguing to notice that the FDM^±^ sits at the frontier between two different topological phases, indicating the closing of a gap between two magnon bands^46^. In light of our present work, and since the topological phase for 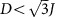 is the same down to *D*=0 (ref. 46), it would be of great interest for future work to study the finite temperature physics of the DM ferromagnet. This is especially true since chiral magnonic edge states and topological skyrmion excitations have been observed in simulations for *D*/*J*∼0.4 (ref. 47).

### Quantum fluctuations

Our analysis has been so far focused on classical spins to precisely determine the nature of their classical ground states. However, it is important to keep in mind that our present threefold mapping is also exact with quantum spins. Indeed the local transformations 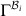 are proper rotations, that is, unitary matrices, and therefore preserve the commutation relations of the spin components. It means that all the mappings illustrated in Fig. 1 can be applied to quantum spins of any size *S*. This important result makes it very tempting to investigate how the previous classical analysis evolves for quantum Hamiltonians.

**Figure 4:**
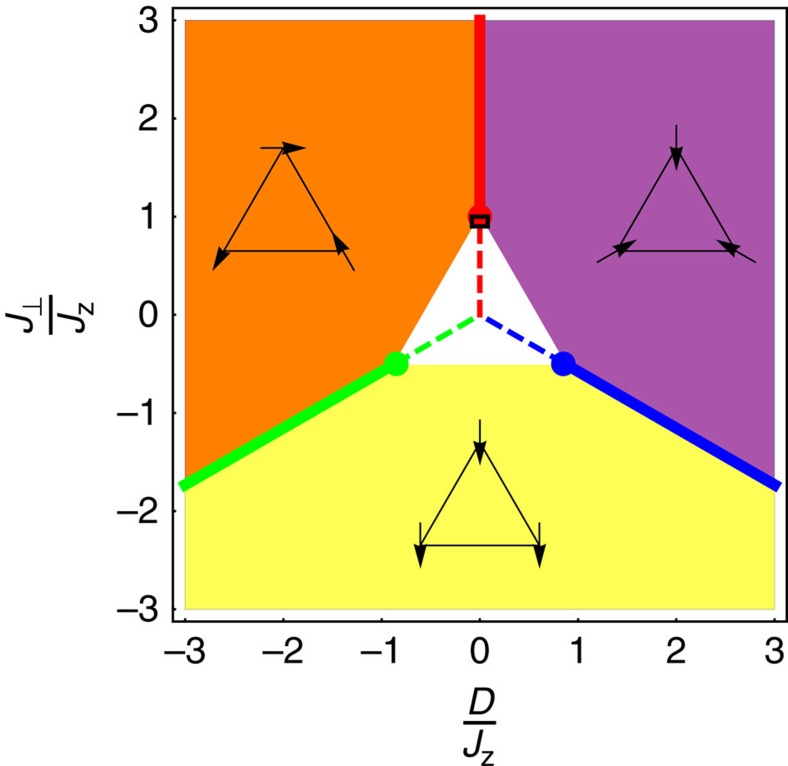
Phase diagram of the anisotropic nearest-neighbour Hamiltonian. This phase diagram is obtained from linear spin wave theory for *J*_*z*_>0. The Heisenberg antiferromagnet and 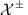 spin liquids are marked by dots and the yellow, orange and purple regions are long-range ordered phases as displayed on the figure. The white triangle delimits a regime where quantum corrections to the order parameters diverge, indicating a possible extended region of quantum disorder. In particular, the density matrix renormalization group method (DMRG) has shown that the entire XXZ model (solid and dashed red lines) is a quantum spin liquid^60^,^61^. Our threefold transformation maps this quantum spin liquid onto the XXZ^±^ models of equation (6) (green and blue lines) for 0<*δ*<1 (solid) and *δ*>1 (dashed), which are thus also quantum spin liquids. Experimentally, independent parametrizations of the Herbertsmithite compound^50^,^51^,^58^ put it at the tip of the white-triangle region (black rectangle).

Let us start by considering quantum fluctuations in the framework of linear spin wave theory. We shall consider the Hamiltonian of equation (1) whose phase diagram is given in Fig. 4 for *J*_*z*_>0. Approaching any of the HAF or 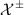 models (marked by dots), the linear spin wave Hamiltonian takes the same form, which simply confirms the equivalence of these three spin liquids in the presence of quantum fluctuations. The linear spin wave calculation also indicates the likelihood of quantum disorder around the centre of Fig. 4. Approaching the white-triangle region, a flat band of excitations collapses to zero energy, leading to a divergence in the quantum correction to the order parameter.

Within the phase diagram of Fig. 4, the *J*_⊥_=*J*_*z*_=*J*>0 line has drawn substantial interest for its relevance to Herbertsmithite ZnCu_3_(OH)_6_Cl_2_, where DM interactions are not negligible (*D*/*J*∼0.044−0.08)^48^,^49^,^50^,^51^. We reproduce the results of refs 31, 52 done on this line of parameters, namely that classically^31^ and up to linear order in spin wave theory^52^, magnetic order is stabilized for any finite value of *D*. However, higher order terms in quantum fluctuations studied by Exact Diagonalization^53^,^54^, Schwinger–boson^55^,^56^ and perturbative methods^57^ have shown that quantum disorder actually persists over a finite region up to *D*/*J*∼0.1, which includes Herbertsmithite. Our goal here is not to claim explanation of the spin-liquid nature of Herbertsmithite which has been extensively studied, but rather to set our theory on an experimental footing. In particular, it should be noted that at linear order in quantum fluctuations, the small XXZ anisotropy observed in Herbertsmithite^49^,^58^ (*J*_⊥_/*J*_*z*_≈0.9) brings this compound at the tip of the white-triangle region with quantum disorder.

Over the past year, the XXZ line with *D*=0 and 0<*J*_*z*_<*J*_⊥_ has also received significant attention for the spin-liquid nature of its ground state for spins *S*=1/2 (refs 59, 60, 61), and the complex quantum order-by-disorder mechanism that takes place for *S*>1/2 (refs 59, 62). Noticeably for spin-1/2, the density matrix renormalization group (DMRG) approach indicates that the quantum spin liquid persists for 0<*J*_⊥_/*J*_*z*_<1 and *D*=0 (refs 60, 61) (see solid and dashed red lines in Fig. 4). The consequences on our work are multiple.

First of all, we had previously shown the extensive nature of the XXZ^±^ classical ground states for *δ*≤1 in equation (6). Our threefold mapping applied to spin-1/2 now makes the XXZ^±^ models quantum spin liquids for all positive values of *δ* (see solid and dashed, green and blue lines in Fig. 4). Furthermore, the central point of our phase diagram *D*=*J*_⊥_=0 has Ising anisotropy and is known for remaining a quantum paramagnet even for arbitrarily small transverse fields^28^,^63^,^64^. As such, it has been described as a rare example of ‘disorder-by-disorder', a mechanism proposed by Fazekas and Anderson^65^, where quantum fluctuations select a disordered sub-manifold of the classically degenerate ground state. Within the framework of our threefold mapping, this remarkable resistance to order can be understood as the consequence of being at the intersection of three (dashed) lines of spin liquids, in a way reminiscent of what has been observed in pyrochlore systems^66^. However, please note that the spin-1/2 phase diagram is expected to be highly anisotropic around this central point, since it has been shown to order into a superfluid phase for *D*=0 and *J*_⊥_<0 (refs 60, 67), and thus also along the symmetric lines 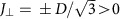 around the origin according to our threefold mapping.

We have discovered a connected network of quantum spin liquids on the kagome lattice, which are mapped onto each other via a threefold transformation. One of the branches of this network is the anisotropic XXZ model, known to be a quantum spin liquid for spin-1/2 (refs 59, 60, 61) (see the red lines in Figs 1 and 4), which includes the actively studied HAF. While every triad of Hamiltonians connected by this mapping have exactly the same energy spectrum at the classical and quantum level, the corresponding spin configurations are necessarily transformed by 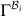. As a consequence, the threefold mapping of the XXZ line gives rise to spin liquids with intrinsic vector chirality because of DM interactions. The Ising antiferromagnet sits at the centre of this map (see Fig. 4), which sheds a new light on the unique propensity of this model to remain disordered^28^,^63^,^64^.

Beyond these three branches of quantum spin liquids, we have studied the stability of Hamiltonian (1) for *J*_*z*_>0, up to linear order in spin wave theory. We have found an extended region of the phase diagram in Fig. 4 where quantum disorder prevails. The small XXZ anisotropy observed in Herbertsmithite^49^,^58^ (*J*_⊥_/*J*_*z*_≈0.9) brings this compound within the tip of this extended region.

At the classical level, the HAF maps onto two models where algebraic correlations take the form of FM pinch points visible in the structure factor of Fig. 2. Keeping the DM term constant, if one tunes the *J*_*z*_ coupling of these models until they become isotropic ferromagnet, the chirality can spontaneously become scalar.

## Discussion

Our work opens a wide range of exciting directions to follow, both theoretically and experimentally. In light of the intense research on the HAF and XXZ models, here we propose two lines of systems with the same energy spectra, but different (chiral) magnetic signatures. With this probe at hand and Figs 1 and 4 in mind, it would be of great interest to look for new insights as one approaches these models and their chiral counterparts from different angles in parameter space (*J*_⊥_, *J*_*z*_, *D*). In particular, the spreading of quantum disorder within the white triangle of Fig. 4 and in its vicinity shall conserve the threefold symmetry, and be mediated by quantum order-by-disorder mechanisms as we vary the spin length *S*^59^,^62^.

The inclusion of 2nd and 3rd nearest-neighbour interactions *J*_2_=*J*_3_=*J*_NNN_ is known to stabilize a chiral spin liquid at finite value 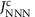 (refs 21, 22, 23, 24, 25, 26, 60, 61, 68). This value 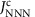 has been shown by DMRG to decrease as the antiferromagnetic *J*_*z*_ coupling vanishes^60^,^61^. This means that the chiral spin liquid is getting closer to the nearest-neighbour XXZ model as *J*_*z*_ goes from 1 to 0. It would thus be very tempting to extend this work to FM coupling (*J*_*z*_<0) towards the XXZ_0_ and equivalent FDM^±^ points. Since 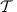 symmetry can be spontaneously broken in the classical FDM^±^ ground states, the possible connection with the chiral spin liquids at finite *J*_NNN_ is a particularly attractive open question.

Beyond kagome physics, the present methodology can be applied to a broad range of lattices and dimensions^69^. Our results especially suggest that systems supporting the ‘disorder-by-disorder' mechanism^65^, such as the Ising antiferromagnet here^28^, are good places to look for hidden spin liquids in the neighbouring parameter space.

On the experimental front, our work fits within the on-going effort for the experimental realization of frustrated systems in optical lattices^29^,^30^, and especially to produce tunable synthetic DM interactions^43^,^44^,^45^.

We hope that our results will further motivate experimental efforts on the synthesis and characterization of kagome materials with anisotropic nearest-neighbour interactions. The recently synthesized ternary intermetallic compounds Dy_3_Ru_4_Al_12_ (ref. 12) and Yb_3_Ru_4_Al_12_ (ref. 13) are very promising materials to start with, since the 4f orbitals of rare-earth ions are known to induce very anisotropic and short-range interactions. Furthermore the presence of itinerant electrons make them natural materials to probe the chirality of the underlying spin texture. Their crystal structure, however, corresponds to a distorted kagome lattice. To impose kagome symmetry is a chemistry challenge, but such was the case for Volborthite Cu_3_V_2_O_7_(OH)_2_·2H_2_O, 14 years ago^6^, which predated the synthesis of a growing number of materials with essentially perfect kagome symmetry^7^,^8^,^9^,^10^,^11^. According to our threefold mapping, some of the places to look for would be large antiferromagnetic *J*_*z*_, as well as around the XXZ_0_ point where no DM terms are required. In light of refs 46, 47, the region neighbouring the FDM^±^ models is also very promising, even for smaller values of *D* and anisotropic *J*_*z*_. At the proximity of these high-symmetry points, especially the one at the centre of the white triangle, chemical, hydrostatic and uni-axial pressure might help the exploration of the phase diagram, as done in rare-earth pyrochlore oxides^70^.

## Additional information

**How to cite this article:** Essafi, K. *et al*. A kagome map of spin liquids from XXZ to Dzyaloshinskii–Moriya ferromagnet. *Nat. Commun.* 7:10297 doi: 10.1038/ncomms10297 (2016).
